# Reconstruction of recurrent shoulder dislocation with glenoid bone defect with 3D-printed titanium alloy pad: outcomes at 2-year minimum follow-up

**DOI:** 10.1186/s12891-023-07148-5

**Published:** 2024-01-02

**Authors:** Danlei Huang, Zhiyang Ye, Jun Wang, Feixiong Chen, Haoyuan Liu, Jianming Huang

**Affiliations:** Department of Orthopedics, Chenggong Hospital of Xiamen University (the 73th Group Military Hospital of People’s Liberation Army), 94 Wenyuan Road, Siming District, Xiamen City, Fujian Province 361000 China

**Keywords:** Recurrent shoulder dislocation, Glenoid bone defect, 3D-printed titanium alloy pad

## Abstract

**Background:**

To evaluate the outcome of shoulder arthroscopy-assisted implantation of three-dimensional (3D)-printed titanium pads for recurrent shoulder dislocation with glenoid bone defects.

**Methods:**

From June 2019 to May 2020, the clinical efficacy of 3D printed titanium pad implantation assisted by shoulder arthroscopy, for the treatment of recurrent shoulder dislocations with shoulder glenoid defects was retrospectively analyzed. The American Shoulder and Elbow Surgeons (ASES) shoulder, Rowe, and Constant scores were recorded before surgery and at 3 months, 6 months, 1 year, and 2 years after surgery. 3D computed tomography (CT) and magnetic resonance imaging were used to evaluate the location of the glenoid pad, bone ingrowth, joint degeneration, and osteochondral damage.

**Results:**

The mean age of the 12 patients was 21.4 (19–24) years and the mean follow-up time was 27.6 (24–35) months. The Visual Analog Scale score significantly improved from 5.67 ± 1.98 preoperatively to 0.83 ± 0.58 postoperatively (p = 0.012). The postoperative ASES score was significantly increased to 87.91 ± 3.47 compared with preoperative ASES score (46.79 ± 6.45) (p < 0.01). Rowe and Constant scores also improved from 22.5 ± 12.34 and 56.58 ± 7.59 preoperatively to 90.83 ± 4.69 and 90.17 ± 1.89 at 2 years postoperatively, respectively. CT performed 2 years after surgery showed that the pad perfectly replenished the bone-defective part of the shoulder glenoid and restored the articular surface curvature of the shoulder glenoid in the anterior-posterior direction, and the bone around the central riser of the pad was tightly united. Magnetic resonance imaging 2 years after surgery showed that the humeral head osteochondral bone was intact, and there was no obvious osteochondral damage.

**Conclusions:**

3D printed titanium pads are a reliable, safe, and effective surgical procedure for treating recurrent shoulder dislocations with glenoid bone defects.

## Background

Shoulder dislocation is a common sports injury, with anterior dislocation being the most common [1, 2]. Approximately 5–32% of anterior dislocations of the shoulder joint are accompanied by a glenoid bone defect, which often leads to anterior instability of the shoulder joint [3, 4]. Recurrent dislocation of shoulder joint with bone defect is regarded as an important factor for failure of shoulder stabilization surgery [5, 6]. Bankart repair of shoulder dislocation has been reported to have a recurrence rate of 4% in patients without significant bone defects and 67% in those with bone defects [7]. When the glenoid bone defect is ≥ 20%, bone reconstruction techniques such as Bristow procedure, Latarjet procedure, and iliac bone graft are usually used [8]. All three surgical procedures were performed to strengthen the stability of the anterior shoulder joint by repairing the bone defect at the anterior edge of the glenoid [9]. Although the efficacy of these three surgical methods has been widely recognized, donor site lesions, nerve injury, bone nonunion, and resorption remain major complications of these bone reconstruction techniques [10–12]. Bristow-Latarjet surgery is a non-anatomical reconstruction technique with high probability of osteoarthritis [13]. Moreover, the Bristow-Latarjet procedure provides a sling effect, but it destroys or does not intentionally preserve the coracoacromial (CA) arch, which may lead to superior instability [14]. Iliac bone grafts are often used to reconstruct glenoid bone defects and repair them after failure of the Bristow-Latarjet operation; however, defects in the donor site, bone nonunion, and bone resorption remain inevitable problems [15, 16]. Therefore, avoiding the shortcomings of the above bone reconstruction techniques and accurately reconstructing glenoid bone defects is still an urgent problem that needs to be studied.

Titanium and its alloys have become popular in the clinic because of their biocompatibility and resistance to corrosion [17]. The application of titanium bone scaffolds in bone-defect repair has attracted wide attention and is considered a feasible method for repairing bone defects beyond the critical defect value. However, traditional titanium alloy implants are stiffer than the cortical bone and may pose a risk of infection and aseptic loosening. An effective approach to reduce the biomechanical mismatch between titanium alloy implants and the cortical bone is to fabricate porous structures. This cannot be achieved using traditional forging or casting techniques but can be performed using three-dimensional (3D) printing technology [18]. In recent years, 3D printed titanium alloy has been widely used for repairing and retreating various types of bone and joint injuries. 3D printed titanium alloy has advantages over traditional bone and joint defect repair materials, such as strong forming ability and short processing cycles and can be tailor-made for each patient. Inspired by 3D printing technology and the concept of artificial joint treatment, this study aimed to evaluate the outcomes of shoulder arthroscopy-assisted implantation of 3D-printed titanium pads for recurrent shoulder dislocation with glenoid bone defects. The application of 3D printing technology can perfectly restore the anatomical shape of the glenoid facet and depth, especially the physiological radius of the glenoid anterior edge, which theoretically avoids the shortcomings of traditional bone reconstruction technology.

## Methods

### General data

The clinical data of 12 patients who underwent arthroscopic “3D printed shoulder cup pad” implantation from June 2019 to May 2020 were retrospectively analyzed. The patients underwent standard medical history inquiry and physical examination before surgery, and were examined using 3D computed tomography (CT) and magnetic resonance imaging (MRI).

The inclusion criteria were as follows: (1) patients who voluntarily provided informed consent. (2) Confirmed diagnosis of recurrent shoulder dislocation accompanied by severe bone defects on the affected side with no deformity, restricted movement, or history of shoulder dislocation on the unaffected side. (3) Patients without any other significant underlying diseases. (4) Preoperative 3D CT scans indicated severe glenoid bone defects in > 20% of the glenoid. (5) Patients experiencing pain and a positive apprehension test during extreme shoulder abduction and external rotation. (6) Patients who had previously undergone unsuccessful shoulder instability surgeries (such as Bankart repair, Latarjet procedure, or iliac crest bone grafting) and experienced recurrent shoulder dislocation.

The exclusion criteria were as follows: (1) patients with multidirectional shoulder instability. (2) Concomitant injuries on the same shoulder side, such as rotator cuff injuries or nerve damage. (3) History of previous surgeries on the affected shoulder, such as rotator cuff repair or acromioclavicular joint dislocation.

All 12 patients were military personnel with a history of > 5 recurrent shoulder dislocations and > 20% glenoid bone defects. All 12 cases were associated with varying degrees of Hill-Sachs injury but no other lesions, such as rotator cuff injury or acromioclavicular joint dislocation. This study was approved by our Ethics Review Committee. All the participants provided written informed consent.

### Design of 3D printed guides and prostheses

**Fig. 1:**
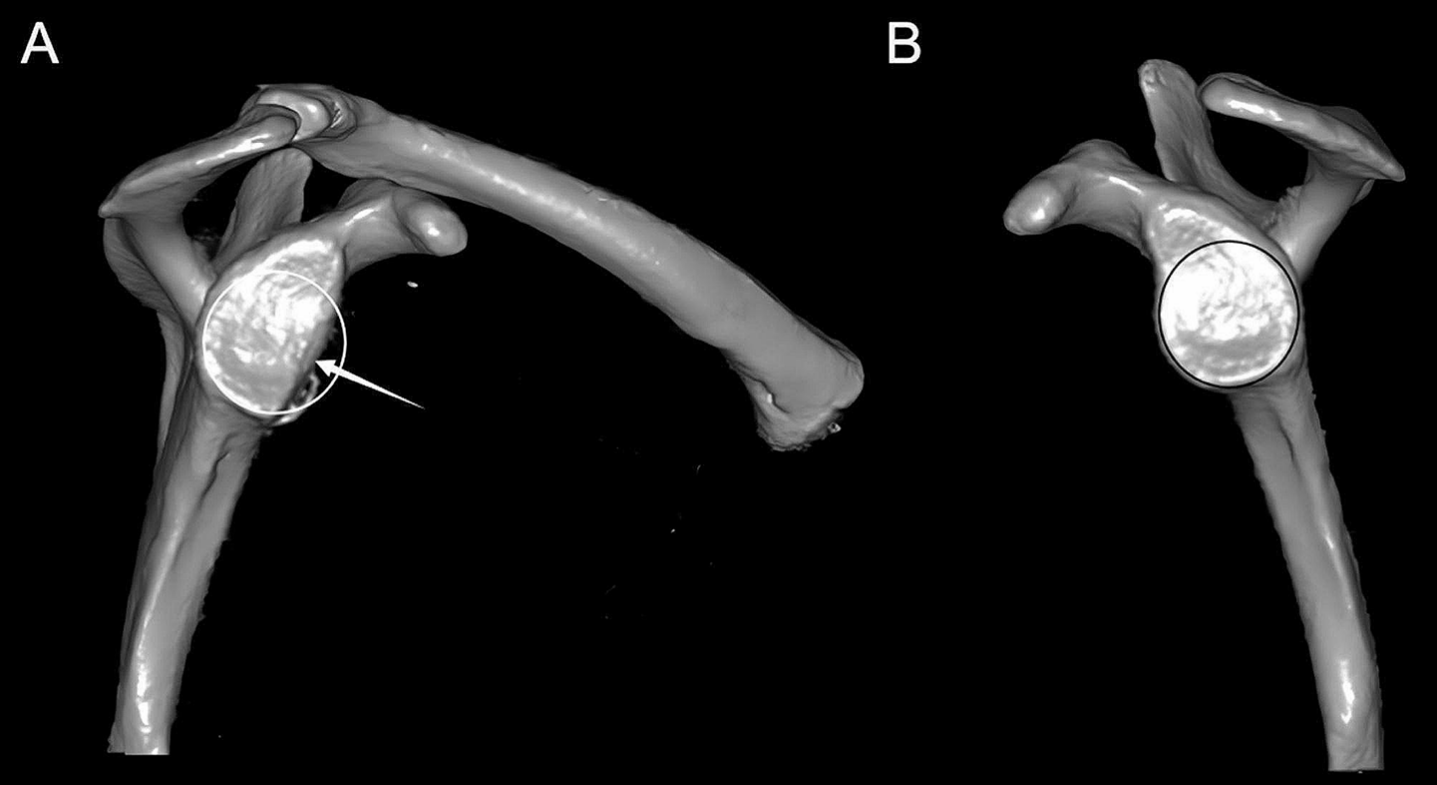
Preoperative 3D CT images of the affected side (**A**) and the healthy side (**B**) in the same case. The arrow indicates 25% anterior glenoid defect

**Fig. 2:**
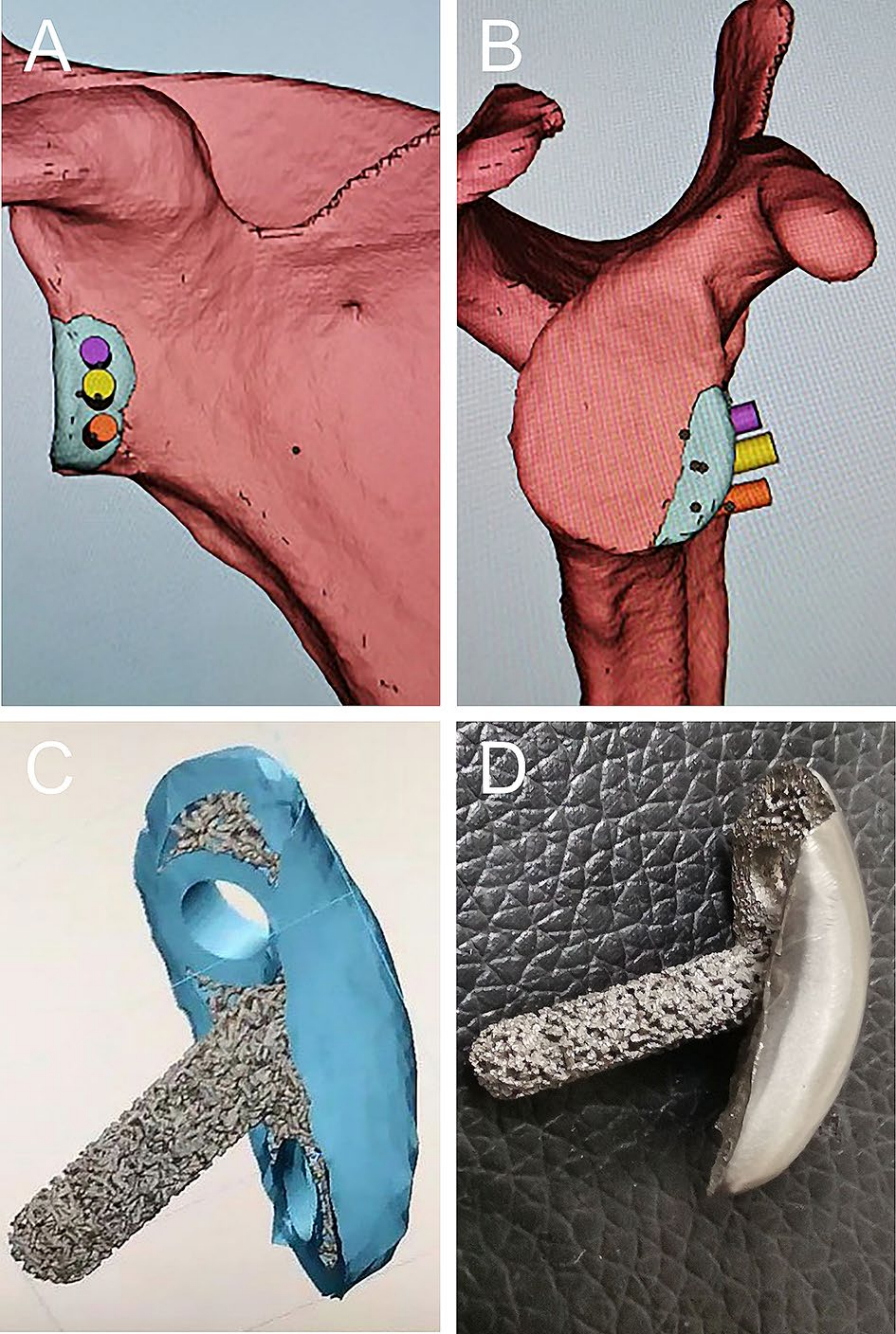
The 3D model drawing and the physical image of the pad. (**A**) A front view of the reconstructed pad model from the 3D simulation. (**B**) A back view of the reconstructed pad model from the 3D simulation. (**C**) The 3D model drawing of the pad. D. The physical image of the pad

First, the degree of the bone defect was evaluated according to the results of a 3D CT examination of the healthy and affected sides of each patient (Fig. 1). Then, the CT scan DICOM data were imported into the Mimics software (version 16.0; Materialise, Leuven, Belgium) for 3D model reconstruction. Simultaneously, the CT data of the contralateral shoulder joint were modeled in 3D, mirror-turned, and compared with the 3D model of the reconstructed shoulder glenoid defect. According to the model, a guide plate and titanium alloy microporous shoulder glenoid cushion prosthesis were fabricated using 3D printing technology. The prosthesis was made of a titanium alloy, and a glenoid pad was printed using an EOS M280 DMLS machine (EOS GmbH, Germany) (Fig. 2).

The 3D printing guide plate had to match the shape of the rotator cuff defect to ensure the uniqueness of the anterior position of the glenoid. The 3D printing guide plate was provided with three kerf needle guide holes, with the middle guide hole located at the three-point position of the rotator cuff and oriented 45° to the longitudinal axis of the rotator cuff to facilitate the positioning of the kerf needle into the rotator cuff through the rotator cuff gap.

The glenoid pad prosthesis consists of a column and articular surface prosthesis. The diameter of the column was 4.5 mm, and the length was individually designed according to the structure of the shoulder glenoid. Screw holes were reserved on both sides of the column for the articular surface prosthesis. After implant placement, two screws were used for further fixation to ensure early stability of the prosthesis. The bone contact surface of the column and articular surfaces of the prosthesis are microporous, which is conducive to bone ingrowth and realizes the biological healing of the prosthesis.

### Surgical techniques

Four surgeons with extensive experience participated in the surgery, with the lead surgeon having > 10 years of experience in shoulder surgery, two surgeons with approximately 5 years of experience, and one surgeon with only 2 years of experience in shoulder surgery.

Patient positioning and arthroscopy: The patient was placed in a beach chair position, and general anesthesia plus brachial plexus block was administered during the operation. All surgeries were performed by the same physician. Shoulder arthroscopy was performed to assess the presence of rotator cuff injury, SLAP injury, degree of bone defect in the anterior shoulder glenoid, Hill–Sachs injury, and occlusal injury between the humeral head and glenoid cavity (engaging).

**Fig. 3:**
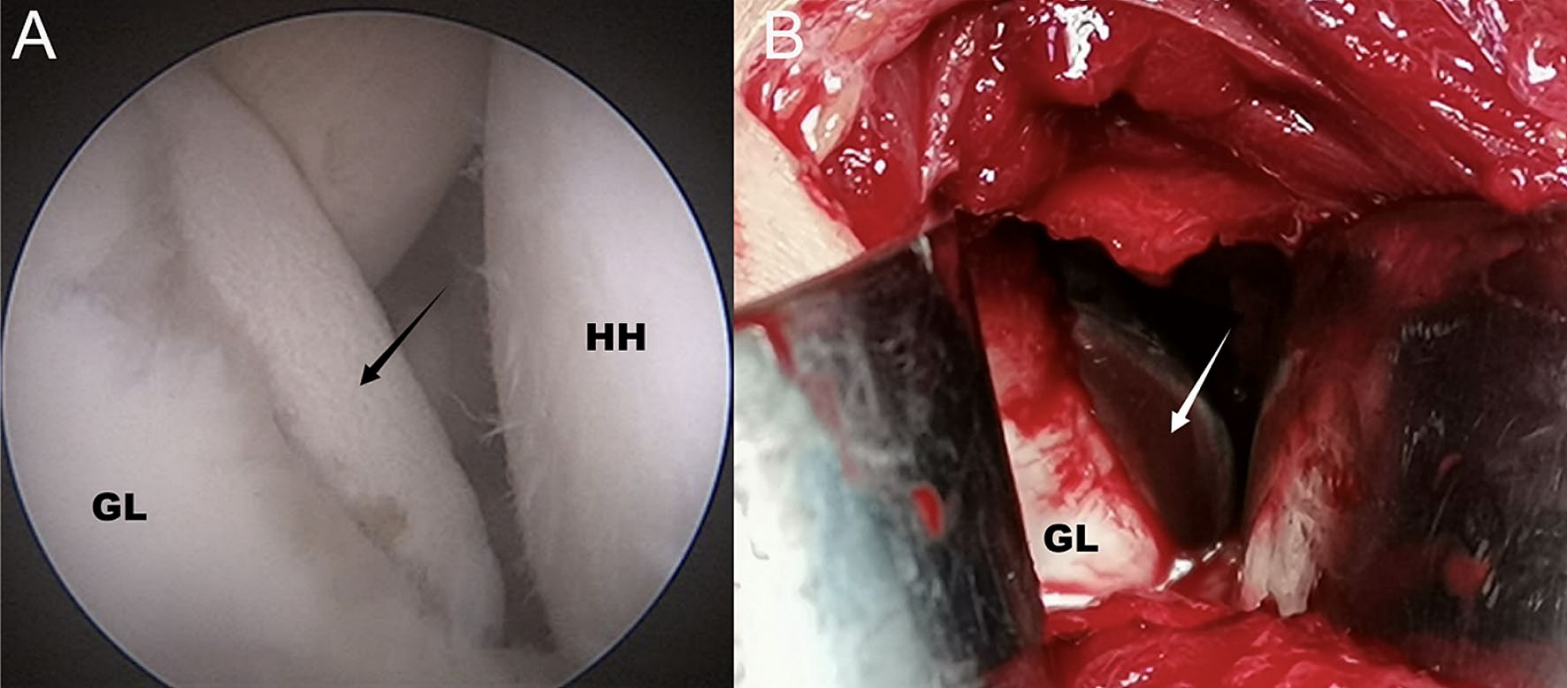
(**A**). The arthroscope is viewed from the posterior approach, and the operator stabilizes the 3D printed guide with finger assistance to ensure proper placement of the guide. The arrow indicates 3D printed guide plate. (**B**) An auxiliary incision of about 4 cm was made anteriorly to the shoulder joint, and the subscapularis muscle was retracted through the rotator interval to expose the anterior bony surface of the glenoid. Under direct vision, the 3D-printed prosthesis was implanted after ensuring that the guide plate was in the correct position. The arrow indicates 3D printed titanium alloy pad. GL, glenoid; HH, humeral head

**Fig. 4:**
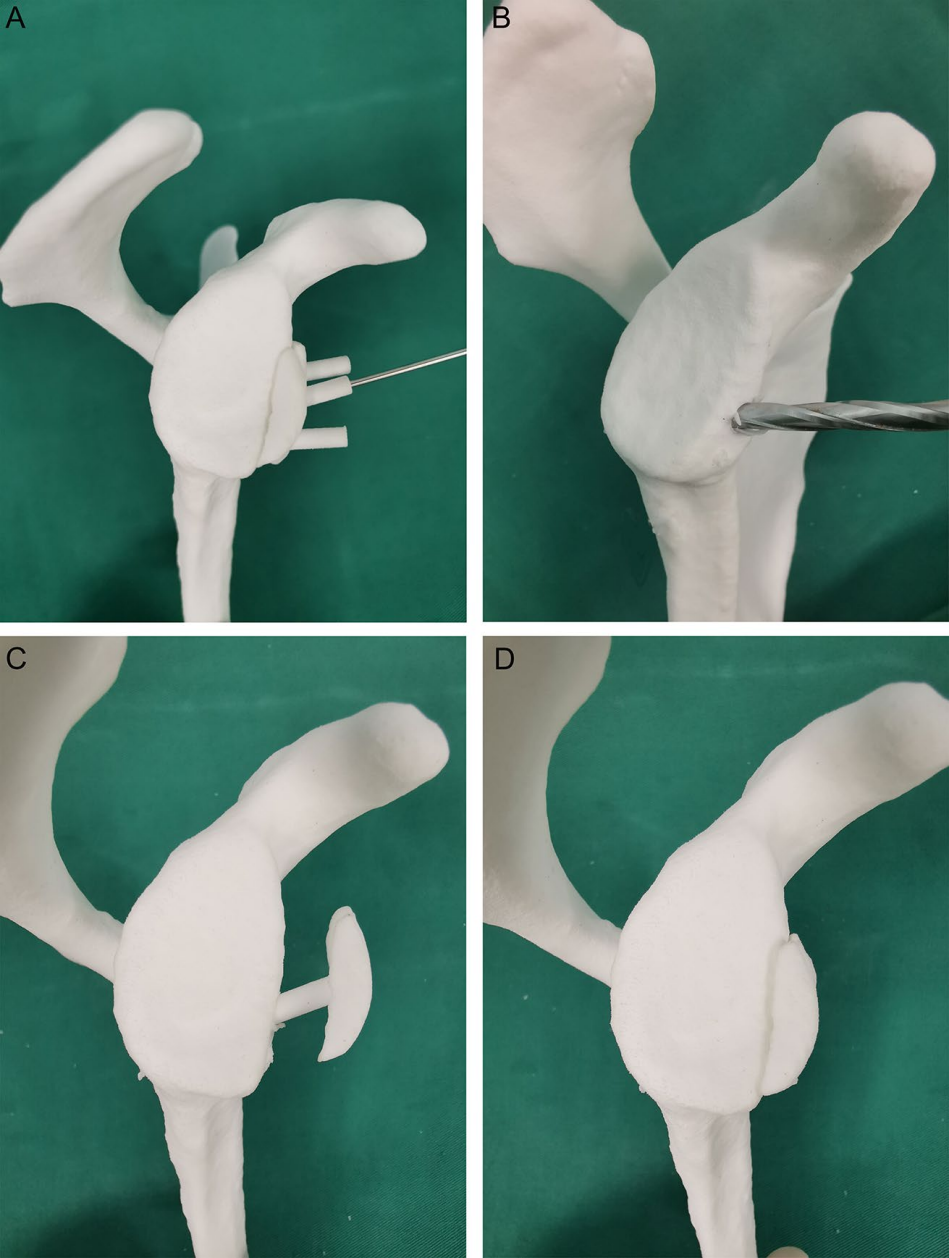
3D printed pad mock-up installation. (**A**) A Kirschner wire was inserted into the middle guide hole of the 3D-printed guide plate. (**B**) The 4.5 mm hollow drill was used to enlarge the bone channel. (**C**) The central column of the pad is driven into the shoulder glenoid through the bone channel. (**D**) Simulation drawing of 3D printing pad after installation

Implantation of 3D printed pads: The rotator cuff space was completely opened, and the coracoid process was exposed under arthroscopy. The anterolateral glenoid labrum and joint capsule tissue were loosened and a three-point position was marked on the cartilage of the anterior border of the shoulder glenoid (right shoulder). A longitudinal incision (approximately 4 cm) was made on the lateral margin of the coracoid process to expose the front of the glenoid, and a 3D-printed guide plate was placed to ensure that the guide plate and the anterior margin of the glenoid were perfectly placed. After observing from posterior approach with an arthroscope, a 2.0 mm Kirschner wire was inserted into the middle guide hole for positioning (usually at the three-point position) (Fig. 3A). After the guide plate was removed, the bone canal was enlarged using a 4.5 mm hollow drill bit, and the bone at the anterior edge of the glenoid was lightly polished. The 3D printed titanium alloy pad was driven in the direction of the bone channel, knocked, and tamped (Fig. 3B). Two holes were drilled in each of the two reserved nail holes on the pad and two 3.0 mm hollow screws were screwed in to reinforce the pad (Fig. 4).

**Fig. 5:**
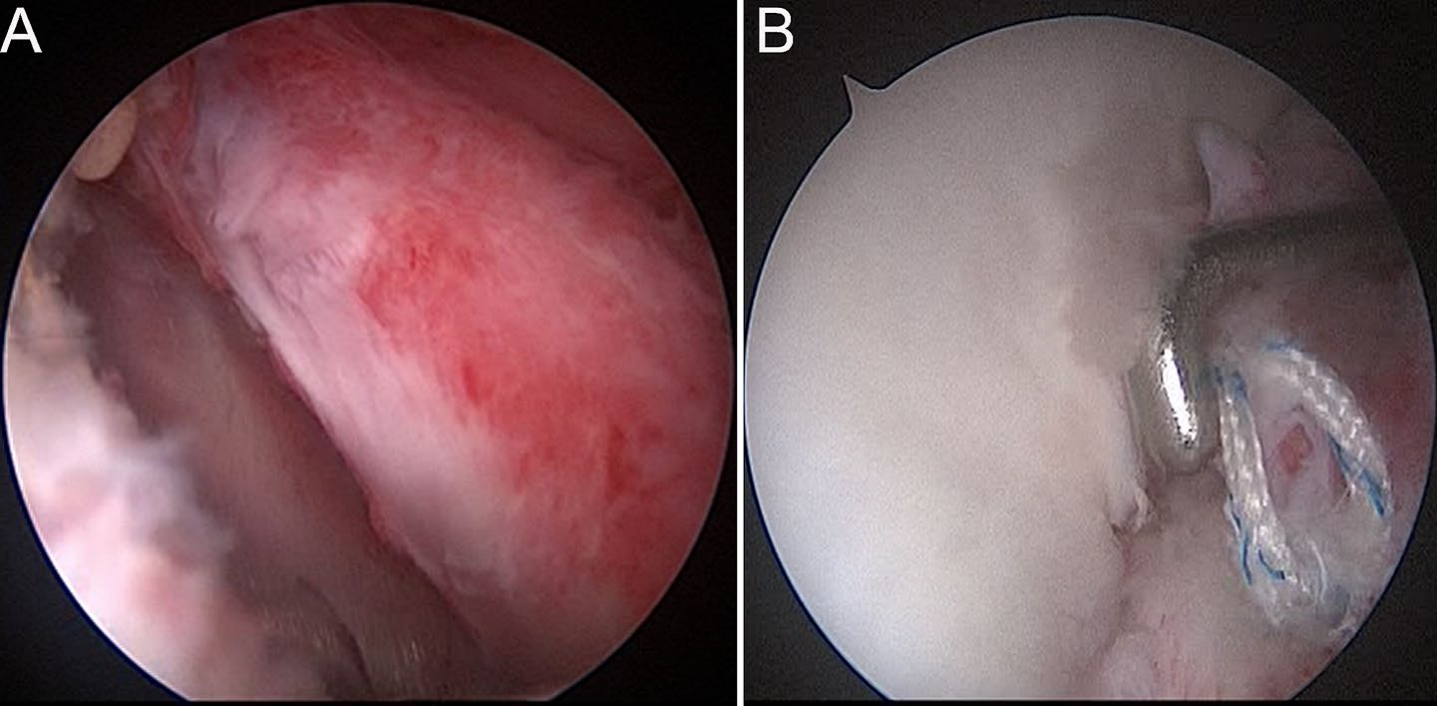
(**A**) Arthroscopically observed from the posterior approach, the 3D-printed prosthesis fit the bony surface of the shoulder glenoid and did not appear to be higher than the articular cartilage surface, and the prosthesis was well positioned. (**B**) Arthroscope from the rear approach, suture the capsule and cover the surface of the prosthesis

Suture of the joint capsule: The arthroscope was again viewed through the posterior channel to confirm the correct placement of the prosthesis (Fig. 5A). Shoulder abduction and external rotation were used to assess shoulder stability. After confirming that there was engagement, anchor nails were implanted at the 4 and 2 o’ clock positions of the shoulder glenoid (right shoulder). The staples were routinely sutured, tied, and fixed; the joint capsule structure was sutured; and the surface of the prosthesis was covered (Fig. 5B).

### Postoperative rehabilitation

Postoperatively, the shoulder joint was immobilized with a shoulder brace for 4 weeks to maintain a neutral shoulder abduction position. Within 4–6 weeks, the patients were encouraged to perform more active elbow, wrist, and hand activities, and shoulder joint activities were limited to passive forward flexion supination, external rotation, internal rotation, and pendulum movements. Active and passive joint mobility exercises and muscle strength exercises were performed after 6 weeks, according to the results of the review. After 8–9 weeks, the patient regained muscle and strength and gradually resumed antagonistic exercise or heavy physical activity 6 months after surgery.

### Preoperative and postoperative evaluations

Clinical outcomes, including visual analog scale (VAS), American Shoulder and Elbow Surgeons (ASES) shoulder, Rowe, and Constant scores, were recorded before surgery, 3 months, 6 months, 1 year, and 2 years after surgery. 3D CT and MRI were performed 1 and 2 years after the surgery to evaluate the position of the shoulder glenoid pad installation, shoulder glenoid pad and bone ingrowth, joint degeneration, and osteochondral damage.

### Statistical analysis

Data were analyzed using SPSS 10.0 and origin software. Data are presented as mean ± standard deviation. A paired t-test was used for comparison between groups, and a p value < 0.05 was considered statistically significant.

## Results

A total of 12 patients with a mean age of 21.4 years (range, 19–24 years) and a mean follow-up time of 27.6 months (range, 24–35 months) were included in this study. All the patients had recurrent shoulder dislocations and shoulder glenoid defects. The basic information of the included patients is shown in Table 1.

**Table 1:** The basic information of the included patients

Information	Median (range) or n (%)
Age	19–24 years (mean 21.4 years)
**sex**	
Male	11/12 (91.7%)
Female	1/12 (8.3%)
Follow up time	24–35 months (mean 27.6 months)
Glenoid defect	20-30% (mean 25.33%)
Hill-Sachs injury	15–20% (mean 17.9%)

The VAS score was significantly reduced from 5.67 ± 1.98 preoperatively to 0.83 ± 0.58 postoperatively (Table 2, p < 0.05). The postoperative ASES score was significantly increased to 87.91 ± 3.47 compared with preoperative ASES score (46.79 ± 6.45), and the difference was statistically significant (Table 2, p < 0.01). The mean preoperative Rowe and Constant scores were 22.5 ± 12.34 and 56.58 ± 7.59, respectively. The postoperative Rowe and Constant scores improved significantly with a mean of 90.83 ± 4.69 and 90.17 ± 1.89, respectively, with significant differences compared to the preoperative data (Table 2, p < 0.05).

**Table 2:** Preoperative and 2 years postoperative rating scores for all patients

Score	Preoperatively	2 years postoperatively	P-value
VAS score	5.67 ± 1.98	0.83 ± 0.58	< 0.05
ASES score	46.79 ± 6.45	87.91 ± 3.47	< 0.01
Rowe score	22.5 ± 12.34	90.83 ± 4.69	< 0.05
Constant-Murley score	56.58 ± 7.59	90.17 ± 1.89	< 0.05

As shown in Table 3, there were no statistically significant differences in shoulder joint mobility between the preoperative and 2-year postoperative periods (p > 0.5). With regard to the postoperative follow-up results concerning shoulder joint range of motion, it is evident that the forward flexion angle averaged 170.83 ± 8.74°, the abduction angle averaged 61.67 ± 6.15°, and the ability to raise a 4.5 kg load was achieved. Such range of motion and shoulder joint strength not only meet the demands of daily activities but also enable engagement in certain upper limb-related physical exercises.

**Table 3:** Preoperative and 2 years postoperative shoulder joint mobility for all patients

	Preoperatively	2 years postoperatively	P-value
Forward flexion	156.67 ± 10.52°	170.83 ± 8.74°	>0.5
Abduction	163.33 ± 15.12°	172.08 ± 6.20°	>0.5
Lateral internal rotation	57.5 ± 7.23°	59.58 ± 6.89°	>0.5
Lateral external rotation	59.68 ± 8.38°	61.67 ± 6.15°	>0.5

**Fig. 6:**
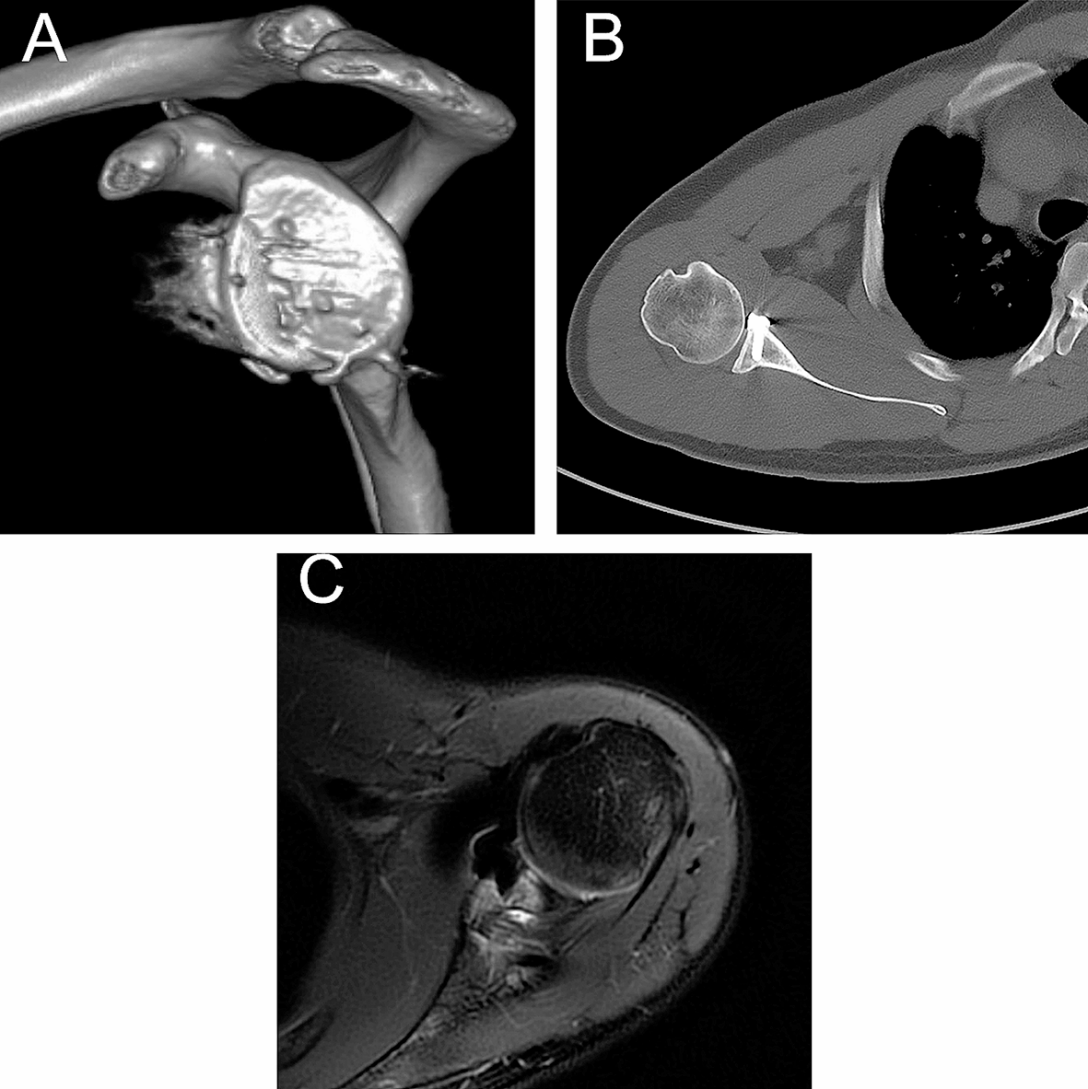
CT and magnetic resonance images of the patient at 2 years postoperatively. (**A**) CT 3D reconstruction 2 years after surgery showed perfect fit of the pad to the defective portion of the shoulder glenoid without malposition. (**B**) CT examination 2 years after surgery showed tight bone union around the pad riser, indicating bone ingrowth. (**C**) Magnetic resonance examination 2 years after surgery showed that the humeral head osteochondral bone was intact without obvious osteochondral damage

The CT 3D reconstruction 2 years after surgery showed that the pad perfectly replenished the bone-defective part of the shoulder glenoid and restored the articular surface curvature of the shoulder glenoid in the anterior-posterior direction, indicating that the pad fit perfectly with the shoulder glenoid defect without misalignment (Fig. 6A). CT also revealed that the bone around the 3D pad column was tightly bonded, indicating bone ingrowth (Fig. 6B). In addition, MRI 2 years after surgery showed that the humeral head osteochondral bone was intact with no obvious osteochondral damage (Fig. 6C).

## Discussion

Recurrent shoulder dislocations are common in clinical practice, and repeated dislocations can easily lead to soft tissue damage and bone avulsion [19]. Approximately 85% of patients with recurrent shoulder dislocations develop glenoid bone defect [20]. When the glenoid bone defect exceeds 20%, it seriously affects the stability of the shoulder joint and easily leads to rotator cuff tears and joint degeneration [21, 22]. Many scholars believe that anatomical reconstruction, which compensates for the lost bone mass of the glenoid and restores the bony structure of the glenoid can restore shoulder joint stability and reduce the recurrence rate of dislocation [23, 24].

Currently, bone surgery methods for shoulder instability mainly include the Bristow procedure, Latarjet procedure, and iliac bone grafting [8]. The Bristow and Latarjet procedures are performed by intercepting the coracoid process and associated tendon, transplanting it to the anterior glenoid bone defect, and fixing it with two screws [25]. The iliac bone grafting is performed by transplanting the bone block cut from the ilium to the glenoid bone defect and fixing it with two screws to repair the glenoid bone defect [26]. These three surgical methods increased the area of the scapular glenoid [27]. However, these surgeries can only restore the width of the glenoid, but not its height, depth, and radian, because the geometric shape of the intercepted bone block cannot completely match the glenoid [28]. When bone resorption and nonunion occur, the anterior stability of the shoulder joint cannot be effectively maintained [29]. In addition, current bone surgeries only repairs the bony structure of the shoulder glenoid and not the glenoid lip. Research showed that the glenoid lip plays a very important role in maintaining the stability of shoulder joint [30, 31]. Gervasi suggested that in surgery for anterior instability of the shoulder joint, it is necessary to use pig skin-derived patches to strengthen the scapular glenoid lip and increase its depth [32]. These surgical procedures also have some disadvantages. For example, the Bristow and Latarjet procedures disrupt the structure of the coracoacromial arch and invade the subscapularis muscle, leading to a high incidence of osteoarthritis [28], and iliac bone grafting is associated with complications, such as avulsion fractures, infection, hematomas, and sensory abnormalities in the donor area [33]. Therefore, new methods for shoulder instability treatment need to be developed.

With the rapid reform and innovation of 3D printing technology, 3D-printed titanium alloy pads are already being developed as devices for repairing bone defects or fractures. It uses computer-aided design software to create a customized shape and size of the pad, and then prints it layer-by-layer with a 3D printer [34, 35]. The 3D printed pads were made of a medical porous titanium alloy, which allowed the model to approximate the shape of the defective shoulder glenoid. Moreover, the porous titanium structure allows for an increased contact area between the implant and host bone, with a modulus of elasticity close to that of human bone [36]. The high connectivity and porosity of the porous titanium structure facilitate the adhesion of osteoblasts, which play a role in bone growth and tissue differentiation, resulting in a higher rate of bone fusion [37]. In this study, we treated recurrent dislocation of the shoulder joint with a severe glenoid bone defect using a biological titanium alloy pad made by 3D printing technology. It not only repaired the glenoid bone defect but also increased the geometric shape of the glenoid, such as width and depth, when used with a pad which exceeded the bone defect width of 2 mm and further strengthened the anterior stability of the shoulder joint. In addition, this technique has no complications at the donor site because it does not involve the body bone tissue and destroys the original anatomical structure of the coracoacromial arch. Our follow-up results showed that the VAS, Rowe, and ASES scores significantly improved compared with those before surgery, and there were no serious complications or obvious osteochondral damage. No obvious bone resorption or nonunion was observed on postoperative imaging. Therefore, the results of our study suggest that when recurrent shoulder dislocation occurs with a severe glenoid bone defect, the biological titanium alloy pad made using 3D printing technology can be used as a reliable treatment.

Loosening and infection are major risks associated with orthopedic implants. Studies have found that in reverse shoulder arthroplasty, insufficient glenoid fixation threatens proper positioning and fixation of the glenoid baseplate, increases the risk of scapular notching and perimeter impingement, premature glenoid component loosening and/or failure, and instability [38, 39]. One meta-analysis reported an overall complication rate of 13.4% after glenoid bone grafting, including iatrogenic nerve palsy, graft nonunion, fracture, hematoma formation, and screw loosening [40]. However, no related complications were found in this study, further demonstrating the safety of 3D-printed titanium pads for the treatment of recurrent shoulder dislocations with glenoid bone defects.

This study had some limitations. First, the number of cases in this group was small, and there may have been biased results in the statistical analysis. Second, the follow-up time of this group of cases was only 2 years, and the long-term results remain unclear. Finally, owing to the inability to mass-produce customized pads, the manufacturing cost is relatively high, which may make it difficult for some patients to afford.

## Conclusion

This study found that 3D printed titanium pads are a reliable, safe, and effective surgical procedure for treating recurrent shoulder dislocations with glenoid bone defects. This technique can reconstruct a glenoid bone defect and restore the width, depth, and geometry of the shoulder glenoid to better maintain the shoulder joint stability.

## Data Availability

The original contributions presented in the study are included in the article, further inquiries can be directed to the corresponding author/s.

## Abbreviations

3D: three-dimensional
ASES: American shoulder and elbow surgeons
VAS: visual analog scale
CT: computed tomography
MRI: magnetic resonance imaging
